# A Computational Pipeline for Retinal Capillary Blood Flow Measurement using Adaptive Optics Line Confocal Ophthalmoscopy

**DOI:** 10.64898/2026.09.21.753337

**Published:** 2026-09-22

**Authors:** Syed Muhammad Hamza Shah, Lin Tong, Ruixue Liu, Yuhua Zhang, Jianhua Wang, Liang Liang

## Abstract

**Background and Objective:**

High-speed and high-resolution retinal imaging using adaptive optics line confocal ophthalmoscopy (AOLCO) resolves individual red blood cells (erythrocytes) flowing through the smallest retinal capillaries, but quantifying their velocity first requires removing linear and nonlinear distortions caused by eye motion that may result in a displacement of tens to hundreds of pixels between frames. We present a computational pipeline that stabilizes AOLCO capillary video and measures erythrocyte velocity in true physical units, delivered as an interactive 3D Slicer extension and a batch command-line tool sharing one analysis core.

**Methods:**

Our video analysis pipeline has four stages: preprocessing, registration, postprocessing, and velocity measurement. During preprocessing, illumination is corrected, and closed-eye frames are identified. During registration, each frame is aligned to a reference frame using affine and, when needed, B-Spline registration, initialized by our robust and fast (GPU-based) translation-registration algorithm to accommodate large eye movements (e.g., microsaccades). During postprocessing, adjacent registered frames are differenced to suppress stationary structures, and a 2D projection of the frame-difference video reveals the capillary network. During velocity measurement, an operator manually identifies capillary segments on the 2D projection image. Spatiotemporal images are then generated along these segments from the frame-difference video, and blood-flow velocity curves are obtained from these images using the Radon transform.

**Results:**

We validate the velocimetry of the pipeline against synthetic ground-truth flow data, and the experiments show that the velocimetry recovers the speed within an error of ±4% The GPU-based translation registration algorithm aligns the large eye motions (∼90 µm per frame on average) and runs about 3.6× (100 Hz) to 38× (400 Hz) faster than the baseline method. Across the full cohort (2344 videos, 57 subjects, 30 Hz to 400 Hz), our registration approach improves structural similarity (SSIM) of the video frames on every acquisition from 0.73 → 0.86. The recovered blood flow velocities are physiologically plausible, of the same order as published AO measurements.

**Conclusions:**

Our computational pipeline enables AOLCO velocimetry on the capillary-level.

## Full Text Availability

The license terms selected by the author(s) for this preprint version do not permit archiving in PMC. The full text is available from the preprint server.

